# Response by Jabbaripour Sarmadian to “Autologous Fat Grafting Combined With Platelet-Rich Plasma in the Treatment of Female Genitourinary Aging: A Retrospective Study” by Ren et al

**DOI:** 10.1093/asjof/ojag108

**Published:** 2026-06-06

**Authors:** Mina Pashaei Ghezeljeh, Amirreza Jabbaripour Sarmadian

We read with great interest the article “Autologous Fat Grafting Combined With Platelet-Rich Plasma in the Treatment of Female Genitourinary Aging: A Retrospective Study” by Ren et al.^1^ They have studied 38 patients with genitourinary aging and reported significant improvements in the external genitalia appearance, features of vaginal aging, genital and sexual function, and urinary symptoms. Similar results were also reported by Lai et al^2^ when they studied micro-autologous fat transplantation (MAFT) technique on 20 patients with vaginal complaints and reported significant improvements in sexual function and dyspareunia, as well as neocollagenesis, neoangiogenesis, and estrogen receptors. Both studies were retrospective with a 6-month follow-up period, so the durability of the observed positive effects is uncertain. The small sample sizes and the limitation of the study populations (patients with vaginal aging and those with vaginal complaints) further affect the strength and generalizability of the findings.

Women of reproductive age frequently suffer from sexual dysfunctions.^3^ However, most women tend to autologous fat grafting (AFG) not because of clinical or vaginal complaints, but rather due to their perceptions and feelings about physical appearance and aesthetic concerns.^4^ This population should be more considered in future studies.

In patients undergoing AFG solely for aesthetic reasons, even at younger ages, we observed potential improvements in sexual well-being and quality of life. Also, the quality of harvested fat appeared to be associated with patients’ dietary habits and physical activity, suggesting a potential link between systemic metabolic factors, graft characteristics, and the primary outcomes of AFG. However, these hypotheses are based on routine clinical follow-up and have not been evaluated within a structured study framework. Further well-designed studies are required to evaluate these observations and explain the underlying mechanisms, as this may be a promising step to solve this common life challenge.
